# Clarifying Misconceptions of the Zone of Latent Solutions Hypothesis: A Response to Haidle and Schlaudt: Miriam Noël Haidle and Oliver Schlaudt: Where Does Cumulative Culture Begin? A Plea for a Sociologically Informed Perspective (Biological Theory 15: 161–174, 2020)

**DOI:** 10.1007/s13752-021-00374-x

**Published:** 2021-02-18

**Authors:** Elisa Bandini, Jonathan Scott Reeves, William Daniel Snyder, Claudio Tennie

**Affiliations:** 1 Department of Early Prehistory and Quaternary Ecology, University of Tübingen, Tübingen, Germany; 2 Department of Human Evolution, Max Planck Institute for Evolutionary Anthropology, Leipzig, Germany

**Keywords:** Cultural evolution, Cultural niche, Cumulative culture, Ratchet effect, Social learning, Zone of latent solutions

## Abstract

The critical examination of current hypotheses is one of the key ways in which scientific fields develop and grow. Therefore, any critique, including Haidle and Schlaudt’s article, “Where Does Cumulative Culture Begin? A Plea for a Sociologically Informed Perspective,” represents a welcome addition to the literature. However, critiques must also be evaluated. In their article, Haidle and Schlaudt (Biol Theory 15:161–174, 2020. 10.1007/s13752-020-00351-w; henceforth H&S) review some approaches to culture and cumulative culture in both human and nonhuman primates. H&S discuss the “zone of latent solutions” (ZLS) hypothesis as applied to nonhuman primates and stone-toolmaking premodern hominins. Here, we will evaluate whether H&S’s critique addresses its target.

## Introduction

Haidle and Schlaudt, in their article, “Where Does Cumulative Culture Begin? A Plea for a Sociologically Informed Perspective” (2020; henceforth H&S), identify three aspects of the “zone of latent solutions” (ZLS) that they critique:

Critique 1 (C1): A critique of any “individualistic” approach to studying culture (which H&S link to the ZLS approach).

Critique 2 (C2): A critique of the individual approach that purportedly requires the existence of fully naive individuals, which H&S argue do not exist.

Critique 3 (C3): H&S further argue that social learning processes other than emulation and imitation play a role in nonhuman animal (henceforth: animal) and premodern hominin (henceforth: hominin) cultures, which they claim is ignored by the ZLS hypothesis.

Here we will discuss how all three of the critiques presented by H&S are erroneous either because they confuse levels of explanation (C1) or because they are based on straw man arguments (C2, C3—but also C1). Notably, in the case of C3, the ZLS hypothesis itself was one of the first, and remains one of the few, to explicitly account for the differential effect of all kinds of social learning mechanisms in animal and hominin cultures. We end with a summary of the growing body of empirical evidence that supports the ZLS account of great ape and hominin culture, contrary to alternative positions.

### Response to C1—Why the Individual Level Cannot be Ignored in Cultural Evolution

H&S criticize the ZLS approach of investigating animal and hominin culture by (mainly) examining the capacities of individual subjects. The authors claim that culture cannot be studied on the individual level because this is an inappropriate atomistic approach. They argue that group-level phenomenon such as culture cannot be reduced down, or perhaps even meaningfully linked, to the individual level. The authors are right to acknowledge the difficulties of linking group-level phenomenon to individual behaviors, but this is neither a new critique, nor is it justified to categorically exclude individual approaches. Research from a variety of fields, including Nobel Prize-winning work in economics, has demonstrated that an understanding of the individual level can be of utmost importance for explaining group-level phenomena (Schelling 1978; Axtell and Epstien 1996; Miller and Page 2007).

The importance of the individual for the group level is further emphasized by the discipline of agent-based modeling (Grimm and Railsback 2012). These modelers are typically interested in the emergent patterns that arise at the group level, and go to great lengths to ensure that behaviors of the individual agents are analogous to the individuals in the systems they study. These models are very relevant to the real world as they inform on the individual behaviors responsible for the group-level phenomenon (Grimm and Railsback 2012). Therefore, the role, capability, and how these individuals behave and interact is paramount to the patterns produced at the group level. Individualistic work^1^ remains important as it produces insight into the breadth and type of behaviors and interactions that produce specific group-level patterns. For example, in Schelling’s classic model of segregation, individual agents who had a slight individual preference for living close to individuals similar to them produced highly segregated neighborhoods at the population level (Schelling 1978). The outcomes of Schelling’s model again emphasize the importance of the individual level for a complete understanding of patterns at the group level. In the absence of information regarding the individual, one may, for example, inaccurately attribute the group-level pattern that arises in Schelling’s model to deliberate decisions. Similarly, the ZLS approach is an empirically founded attempt to examine the individual-level processes that may produce group-level phenomena (such as wild ape cultural patterns; Acerbi et al. 2020).

Further justification for the need of an individualistic approach can be found within H&S’s own arguments. Despite the fact that the authors imply that one must take an exclusively group-level approach, they discuss in depth what individuals are capable of absorbing from the surrounding environment. For example, their notion of habitus (see below) is predicated on having an understanding of the cognitive capacity of the individual. This is not in contrast to what the ZLS attempts to do. H&S go on to provide evidence from experiments of individual rats raised in different conditions to show that behaviors that may appear to be instinctive (see below) are influenced—on the individual level (via specific environments).

Even though we advocate for the individual level, we have never denied the importance of the group level—indeed group-level patterns are also an explanatory target of the ZLS, and were so from the start (Tennie et al. 2009). Clearly, understanding the interplay between the individual *and* group level remains a valid and necessary line of research, despite claims to the contrary.

### Response to C2—The Instinct Debate Does Not Apply to the ZLS Hypothesis

H&S resurrect an old criticism of a concept of instinct—in particular one that sees instincts as behaviors that are innate and fixed. Of course, we (and most, if not all, modern scientists) agree with criticism of this concept. However, we take issue with H&S’s misattribution to the ZLS hypothesis the idea that the zone of latent solutions consists of such instincts.^2^ Indeed, both the term and concept of instinct have been explicitly excluded from the ZLS (labeled as “outdated”; cf. Bandini and Tennie 2018). Latent solutions are not instincts. Instead, latent solutions are behaviors and/or artifacts whose specific forms derive from a complex interplay of factors involving, among other things, both genetic and environmental factors—and even complex cognition is included in this system of interactions.^3^ What makes these forms special instead is that form-copying variants of social learning (e.g., imitation) are not required for them to reappear. This is the crux of the ZLS hypothesis that has been clearly and consistently described in all ZLS publications (e.g., Tennie et al. 2009, 2012; Reindl et al. 2016; Bandini and Tennie 2017, 2018, 2019).

H&S’s confusion regarding the ZLS and the outdated instinct concept can perhaps be linked to their claim that the ZLS hypothesis—and identification of latent solutions—requires tests on fully naive individuals. This claim is incorrect. Perhaps it derives from an overintellectualization of a clearly-labeled thought experiment: the cultural island test (Tennie et al. 2016, 2017). Note that the problems with this particular thought experiment are well known. Indeed, even the first version of the island thought experiment as proposed by Tomasello (1999) paid tribute to these complications. Note that in presenting his island test idea Tomasello specified two things to counter similar objections: (1) A social cohort grows up on the hypothetical island (i.e., sociality is added; Tomasello 2018); and (2) the cohort is “magically kept alive” (implying, amongst other things, emotional, caloric, and antipredator support; Tomasello and Rakoczy 2003). Whilst even this test cannot be run, if it were possible and these forms emerged under these conditions, it would constitute a valid test for subjects’ abilities to *individually* produce forms (i.e., actions and/or artifact topographies that can be organized in a linear or hierarchical structure; Tennie et al. 2020a, b).

What H&S fail to acknowledge is that the practical impossibility of creating an island test does not mean that information relevant to the underlying question cannot be gleaned. Mendel did not see genetic inheritance, but this did not stop him from investigating meaningful inheritance patterns. To detect patterns and factors in culture, approximations and triangulations remain informative. For example, any spontaneous reoccurrence (“reinnovation”) of the same behavioral and/or artifact forms in culturally independent populations (in H&S’s terms different “habitus”^4^) needs to be explained. Reinnovations in species that do not copy behavioral and/or artifact forms require even further explanation (see below).

Empirically, this situation arises when captive, ecologically relevant apes reinnovate wild ape behavioral forms (e.g., Neadle et al. 2017; Bandini and Tennie 2017, 2019,5), because these apes do not copy (Tennie et al. 2009, 2012; Clay and Tennie 2017). We maintain that the ZLS approach is the best explanation for this form reinnovation in apes. Again, why and how these forms and not others are reliably produced is a complex but separate issue (Tennie et al. 2009, 2020a, b). However, whether one particular factor (form-copying) is present, or even required, is a key question for the ZLS. None of this is to say, however, that social learning of other types plays no role in ape culture, but these types merely regulate frequencies of form (a point discussed in greater detail in the following section; e.g., Bandini and Tennie 2017, 2018, 2019; Neadle et al. 2017, 2020; Reindl et al. 2018). However, it remains likely that ape social learning does not transmit behavioral and/or artifact forms.

H&S argue that social learning and social life influence ape and hominin cultures. Both of these ideas are incorporated into the ZLS approach (e.g., Henrich and Tennie 2017; the same is true, of course, of humans). H&S specifically claim that ape and hominin behavioral and/or artifact forms depend in some way on these two factors. No one disagrees that this must be true at some point in hominin evolution, but to us, the question remains when this happened (see Tennie et al. 2016, 2017; see also below). In other words, this cannot be assumed to be true for all species of apes and hominins, as showcased by modern apes. If social life is so important for apes, how come the same forms appear across different populations, including captive individuals (e.g., see footnote 4 and Tennie et al. 2008; Bandini and Tennie 2017, 2019; Neadle et al. 2017,6)?

Therefore, empirically, the two factors proposed by H&S do not prove of importance for these forms in apes. This conclusion also raises serious doubts on H&S’s just-so storyline of hominin culture, given their over-attribution of form dependency on other factors. H&S neglect to acknowledge the empirically supported possibility of form copying-*in*dependent reinnovations (Bandini and Tennie 2017, 2019; Tennie et al. 2016, 2017). Yet, strong arguments can also be made for the application of the ZLS to the archaeological record. Tennie et al. (2016, 2017) point out that there are certain aspects of early stone tools that appear consistent with the ZLS (e.g., arguments on stasis; Semaw 2000). These observations sit very uncomfortably with the alternative hominin storyline proposed by H&S.

Consequently, it is neither the case that the ZLS hypothesis hinges on outdated concepts of instinct, nor does it require real-life applications of any island test setup. The instinct concept remains unnecessary to explain recurrent behavior or artifact forms that do not require form copying. The ZLS has always focused on individual (including cognitive) explanations of form innovative abilities. Various genetic and environmental settings across ontogeny, rather than cumulative cultural evolution and instinct, create and shape the resulting forms of the latent solutions at the individual level (see above).

### Response to C3—Non-Copying Social Learning is an Essential Part of the ZLS

H&S further claim that the ZLS hypothesis fails to address the fact that social learning mechanisms other than imitation and emulation can play a role in behavioral repertoires. H&S promote their preferred *habitus* view as a “novel” alternative—supposedly standing out by incorporating these effects instead. However, even a cursory reading of the ZLS literature demonstrates that these factors are recognized components of the ZLS.

The important role of non-copying social learning mechanisms is a fundamental aspect of the ZLS hypothesis and has been emphasized in every ZLS publication^7^ (Call and Tennie 2009; Tennie et al. 2009, 2010a, b, 2012, 2016, 2017, 2020a, b; Tennie and Hedwig 2009; Hanus et al. 2011; Acerbi et al. 2012; Pradhan et al. 2012; Menzel et al. 2013; Hopper et al. 2015; Acerbi and Tennie 2016; Reindl et al. 2016, 2018, 2020; Tennie 2016, 2019a, b; Bandini and Tennie 2017, 2018, 2019; Clay and Tennie 2017; Henrich and Tennie 2017; Neadle et al. 2017, 2020; Bandini and Harrison 2020; Bandini et al. 2020a, b; Neldner et al. 2020; Tennie and van Schaik 2020; Motes-Rodrigo and Tennie, resubmitted). This should not be surprising, considering that the idea that non-copying social learning mechanisms^8^ play a role in the *frequency* of animal behavioral forms has been an established fact in the animal culture literature for decades.^9^ Therefore, H&S’s third critique ignores the core concepts of the ZLS hypothesis, creating a straw man argument.

H&S’s “alternative” concept of habitus merely repeats the ZLS concept of “socially mediated serial reinnovations” (or SMSR/SMR) originally described in Bandini and Tennie (2017). The overlap between these two concepts forces us to imagine that some unintentional plagiarism occurred here, as when describing their habitus concept, H&S speak of “a zone of socially induced latent solutions” (p. 11), which is, in essence, the original “socially mediated serial reinnovations” of the zone of latent solutions (Bandini and Tennie 2017)^10^—note, “reinnovations” here are latent solutions by definition. Since 2017, the original SMSR concept has been discussed in every subsequent ZLS publication, including the two cited by H&S (Bandini and Tennie 2017, 2019).

H&S also call for a “new” concept of culture in which any social learning effects (of any kind) should be considered cultural.^11^ Again, this is not a new concept. Equating any variant of social learning with culture is a concept that stems from the ZLS literature itself (i.e., Neadle et al. 2017).^12^ Culture, but not cumulative culture of forms, is widespread and the ZLS is and always was a strong proponent of this view.

### A Comparison of Approaches

The descriptions and the terminology underlying the ZLS account have evolved since its first basic description in the literature in 2009 (Tennie et al. 2009). However, the core principles of the ZLS hypothesis have not changed. The “ZLS-only” approach (Reindl et al. 2018) applies to any species that do not copy *know-how* (i.e., do not copy the specific forms of behavior or artifacts; Tennie et al. 2016, 2020a, b). These species do, however, also show know-how—but the forms of these know-hows must derive on an individual level as they are not copied. The ZLS does not deny that atypical upbringing can limit the reach of individual know-how (e.g., in deprived subjects; Henrich and Tennie 2017). On the other hand, extensive human training and rearing may transmit (“install”) form-copying abilities to species that otherwise do not copy forms (installing form-copying as so-called “cognitive gadgets,” sensu Heyes 2018,13). As a result, the affected species may then show additional forms, after first being enabled to copy these forms (see Tennie 2019a; Henrich and Tennie 2017). Yet, empirically, in apes, these cognitive gadgets—once transmitted to the (currently) best possible level—are not self-supporting across generations of apes (Tennie 2019b). In sum, the current natural tendency of apes is to remain a ZLS-only species.^14^

The ZLS-only account has been applied to several species, in particular nonhuman great apes (see summary in Tennie et al. 2020a, b) and early hominins (Tennie et al. 2016, 2017; Tennie 2019a, b). The currently available data suggests that the ZLS-only approach is a good empirical fit for these species. In particular, ecologically representative apes do not show convincing evidence for copying know-how that is out of their cognitive reach (Tennie et al. 2009; Clay and Tennie 2017). Instead, these apes create know-how individually in the absence of models to copy (Allritz et al. 2013; Menzel et al. 2013; Bandini and Tennie 2017, 2019; Neadle et al. 2017). Furthermore, the specifics of the behavioral forms that are reinnovated individually do not differ between populations. This last point is especially important in the current context, as it creates problems for H&S’s argument that all culture is cumulative (see H&S’s abstract). Of course, it is possible that new data might surface supporting the alternative notion, namely that the ZLS-only account is not a good fit for all behavioral and/or artifact forms of all these species. If this evidence is sufficiently strong, it will turn the affected species into a ZLS-plus species; i.e., one that has a ZLS but can also copy forms (Reindl et al. 2018).^15^ Yet, given all that is currently known about nonhuman apes and early hominins (and the LCA), any level of form-copying will likely be very low, both in frequency and in reach.

It may be helpful to delineate the various theoretical positions at stake. In one reading of H&S’s position (Reading 1), culture should always be cumulative specifically with regards to the forms produced^16^—but the fact that specific different cultural backgrounds are not causally linked to the production of specific, *dissimilar* forms in apes (see above) empirically discounts this possibility (instead, different backgrounds nevertheless lead to similar forms). In Reading 2 of H&S’s account, innovations that have already been produced in a population catalyze further, similar, innovations (all still in the absence of copying of know-how). An ongoing process of these socially mediated reinnovations can then also stabilize innovations on a population level, leading to group-level effects. While this is most certainly true, it is part and parcel of the original formulation of the ZLS concept (Tennie et al. 2009) and all following ZLS publications, and therefore is not a novel framework. Reading 2 is essentially the basic ZLS concept. In Reading 3, H&S imply that there are forms that do not need to be copied but are individually reinnovated instead (induced by socially mediated reinnovations) *before* additional, at least somewhat dissimilar, forms can be individually reinnovated (an “internal ratchet effect” of sorts).^17^ This is a theoretical possibility, but is again one that the ZLS approach acknowledges (we call this the “grey zone of cumulative culture”; see Tennie et al. (2020b).^18^ In Reading 4, H&S allow not only social learning of the types already included in the ZLS account, but additionally allow the copying of know-how (i.e., form-copying.^19^ In this reading, it would indeed be expected that the resulting forms themselves can ratchet. However, this would not be a new interpretation, either (indeed, it would be the standard view of cultural evolution; see e.g. Tennie et al. 2017). Lastly, in Reading 5, H&S would be committed to all aspects of Reading 4, but, in addition, acts of form-copying can additionally also change individuals’ innovative abilities (e.g., copied know-how breeds further know-how). Yet, again, this concept would not be new, it would be cumulative cultural intelligence (CCI). Variants of CCI have been around at least since the time of Vygotsky (who described CCI in the so-called zone of proximal development (or ZPD)^20^. Furthermore, we suspect (Tennie et al., 2020a) that the effect of CCI regarding know-how depends to a considerable extent on form-copying. Given that form-copying is absent (or at least nearly absent) in apes, and that it was likely also absent in premodern hominins, Reading 5 is not a good empirical fit for either (as we have highlighted across several publications and above).

Note that there is another possibility,^21^ namely one in which not only the innovative skills of individuals are under some social learning control, but additional cognitive skills are also under social learning control. In this account (recently dubbed *cultural evolutionary psychology (CEP)*; Heyes 2018), even form-copying skills may be socially transmitted (Heyes 2018). CEP is an attractive account for the evolution of cumulative culture in our species—not least due to a large amount of evidence for it in general (Heyes 2018), but also specifically given what is known about apes and early hominins, and the effects of human training and enculturation on apes within and across generations (Tennie 2019a, b). Yet, even in this account, the onset of cumulative culture would have been late in our lineage (namely sometime(s) in the last half-million years; Tennie 2019a, b). In other words, the ZLS hypothesis for apes and premodern hominins is fully compatible with a CEP approach.^22^

## Conclusion

This response has addressed H&S’s three main critiques of the ZLS hypothesis. We have demonstrated that: (1) Both the individual and group levels and their interactions are important. (2) The ZLS does not hinge upon outdated concepts of instincts, unrealistic testing conditions, or totally naïve individuals. (3) The ZLS hypothesis is the origin of the idea that culture equates with any social learning and that non-copying social learning plays an important role in mediating the frequency of form reinnovations (i.e., socially mediated reinnovations). We hope now that the core principles of the ZLS have been re-clarified, we can continue to have fruitful discussions and move the field of cultural evolution forward.

## Footnotes

1 Note that the ZLS’s principal aims explicitly include explaining group-level phenomena (Tennie et al. 2009), and this work includes using agent-based modelling (e.g., Acerbi et al. 2020).

2 “Instinctive behavior has much in common with individual latent solutions, in particular that they are (partly) hereditarily determined and appear in organisms raised in isolation from others” (p. 166 in H&S).

3 Haidle and Schlaudt themselves acknowledge early in their own piece that the ZLS includes cognition.

4 This, in turn, should instead have led to different behavioral forms in H&S’s account.

5 See also Bandini et al. (2020b) for a case of an individual reinnovating a behavior outside of their species-known repertoire in the wild.

6 See also Neldner et al. (2020) for how even a human (baseline) ZLS (Reindl et al. 2016) can be meaningfully studied with a triangulation approach.

7 For example, see Bandini and Tennie (2017, p 16): “In no way does our data negate a role of low-fidelity social learning [i.e. non-form-copying social learning mechanisms] in scooping, or any other chimpanzee behaviour when looking at the population level [sic!]. Indeed, (low-fidelity) social learning mechanisms likely homogenise the likelihood of individual learning of many chimpanzee behaviours, and therefore[…]can play a decisive role in explaining the distribution of these behaviours as they are observed in ape populations.”

8 Also known in the literature as "low-fidelity social learning"; Tennie et al. (2020a).

9 E.g., see also Schuppli and van Schaik (2019), which the authors cite throughout as a counter-stance to the ZLS (which, again, is a false juxtaposition as the two approaches are compatible, and indeed van Schaik is a co-author on two recent publications on the ZLS; Tennie et al. 2020a, b).

10 SMSR are described as: “The underlying mechanism that drives the acquisition of behavioral forms in chimpanzees (and other apes) is that of ‘socially‐mediated serial reinnovations’ (SMSR; Bandini and Tennie 2017). These behavioral forms increase in frequency in populations due to low‐fidelity social learning helping others reinnovate the behavior on their own” (Bandini and Tennie 2019, p. 3).

11 H&S (p. 165; italics in original): “The important point is that we do not ask for the respective *share* of nature and culture in given traits, but rather whether social learning is involved *at all*. If so, the trait counts as cultural, not individual.”

12 Neadle et al. (2017, p. 12): “a behaviour can be considered cultural, if social learning […] plays any role at all in the form and/or the frequency of the behaviour.”

13 Note that this may be true for humans, too—we might also require cultural transmission of copying skills (Heyes 2018). However, in contrast to the other apes, these skills in humans remain consistent enough across generations—enabling a ratcheting of cognitive gadgets in humans (Tennie 2019a). When (and how often) this cognitive gadget ratchet first started is currently an open question (Tennie 2019a).

14 The phylogenetic implication for the LCA is that they were ZLS-only species too (Tennie 2019b). Another implication is that data from enculturated / trained apes (such as, e.g., Kanzi, the bonobo; Toth et al. 1993) cease to be phylogenetically informative (compare also Henrich and Tennie 2017).

15 The paradigmatic case is humans, who often copy even complex forms.

16 It has long been known that other variants of culture (e.g., social learning about locations, or “know-*where*”; Bandini et al. 2020) can evolve, too [what Tennie et al. (2009) called step-wise traditions]. Yet, empirically, these kinds of cultures—widespread in the animal kingdom—do not hold the key to the kind of cultural effects seen in human culture. The human case logically requires form-copying mechanisms (Tennie et al. 2012, 2020a).

17 Or, more generally, where past innovations change the likelihood of future innovations within individuals, and, due to the effects of socially mediated reinnovations, also in affected populations (see Tennie et al. 2020b).

18 This chapter has been in press since 2017 (which is standard for book chapters). Note that the empirical evidence for this gray zone of cumulative culture is very limited in apes—rendering a “ZLS-only” gambit (Reindl et al. 2018; Tennie et al. 2020a) still the best overall empirical fit for the available data.

19 “[H]abitus includes general problem perceptions and implicit knowledge about specific resources, their qualities and application, timing and pattern of acquisition, pathways, locations, and tools as well as *practices of their manufacture, use*, maintenance, and discard” (p. 169 in H&S; emphasis added).

20 Indeed, the ZLS is designed, also by name, as the missing baseline precursor of Vygotsky’s ZPD (Reindl et al. 2018).

21 Note that this specific possibility is incompatible with the H&S account (it is, therefore, not a possible reading of H&S’s piece).

22 It does not matter for the ZLS approach exactly how form-copying is enabled (e.g., via evolution acting on the genetic and/or the cultural level). What matters is whether form copying is enabled (and where form copying *is* enabled, forms can potentially begin to culturally evolve—a mechanism that can make a species escape its ZLS).
